# Antenatal glycemic control in gestational diabetes and its association with postpartum glucose intolerance and weight retention

**DOI:** 10.1016/j.clinsp.2026.101051

**Published:** 2026-07-18

**Authors:** Yue Jiang

**Affiliations:** Endocrinology Department, Qixia District Hospital, Nanjing, Jiangsu, China

**Keywords:** Gestational diabetes mellitus, Antenatal glycemic control, Postpartum glucose intolerance, Postpartum weight retention

## Abstract

**Background:**

Evidence linking antenatal glycemic control to postpartum dysglycemia and weight retention in real‑world GDM care is limited. The authors tested whether late‑pregnancy control predicts early postpartum glucose intolerance and 12‑month Postpartum Weight Retention (PPWR).

**Methods:**

In this single‑center prospective cohort, women with GDM were enrolled from May 2021 to April 2024. Antenatal control (28–36 weeks) from routine SMBG was summarized as Time‑In‑Target (TIT). Mean fasting/postprandial glucose were recorded. The primary outcome was postpartum glucose intolerance by 75 g OGTT at 6–12 weeks, and secondary outcomes were PPWR and substantial PPWR (≥ 5 kg) at 12-months. Multivariable logistic and mixed‑effects models adjusted for prespecified confounders.

**Results:**

Of 348 enrollees, 287 (82.5%) completed the postpartum OGTT and 267 (76.7%) had 12‑month weight. Postpartum glucose intolerance occurred in 82/287 (28.6%). Each 10% lower TIT was associated with higher odds of dysglycemia (adjusted OR [aOR = 1.24], 95% CI 1.10–1.39). The poorest‑ vs. best‑control quartiles had aOR = 2.15 (95% CI 1.31–3.53). Mean PPWR at 12-months was 3.4 ± 4.1 kg; each 10% lower TIT corresponded to +0.32 kg PPWR (95% CI 0.12–0.52) and increased odds of substantial PPWR ≥ 5 kg (aOR = 1.13, 95% CI 1.02–1.25), with Q4 vs. Q1 aOR = 1.92 (95% CI 1.11–3.33).

**Conclusions:**

Poorer antenatal control was independently associated with postpartum dysglycemia and greater 12‑month weight retention, supporting risk‑stratified postpartum OGTT follow‑up and early lifestyle support.

## Highlights

• Antenatal glycemic control predicted postpartum glucose intolerance.
• Lower time-in-target was linked to greater postpartum weight retention.
• Poor control doubled dysglycemia risk versus best-control quartile.
• BMI modified the association between glycemic control and dysglycemia.
• SMBG time-in-target may help prioritize postpartum OGTT follow-up.

## Introduction

Gestational Diabetes Mellitus (GDM) is a prevalent condition affecting 2.4% to 25% of pregnancies globally, with significant regional variations due to differing diagnostic criteria and increasing trends in obesity and maternal age.^1^, ^2^, ^3^ The condition is characterized by glucose intolerance first recognized during pregnancy, often due to pancreatic β-cell dysfunction and insulin resistance.^4^^,^^5^ Short-term risks for mothers with GDM include hypertensive disorders, cesarean sections, and fetal macrosomia, while long-term risks are notably significant, with a 70% chance of developing Type 2 Diabetes Mellitus (T2DM) within a decade postpartum, as well as increased risks of cardiovascular disease and metabolic syndrome.^1^^,^^3^^,^^6^ Despite the importance of postpartum glucose testing to monitor these risks, completion rates remain low, exacerbating the potential for rising Postpartum Weight Retention (PPWR) and cardiometabolic risk.^6^ The postpartum period is critical for lifestyle modification and metabolic surveillance, yet many women do not receive adequate follow-up care.^1^^,^^6^ Improved postpartum screening and targeted interventions therefore remain a public-health priority for reducing the long-term health burden on both mothers and their children.^1^^,^^4^

Limited prospective data indicate that the quality of antenatal glycemic control significantly influences early postpartum glucose intolerance and Postpartum Weight Retention (PPWR) trajectories. Research shows that women with GDM exhibit varying risks of postpartum glucose intolerance based on the severity of their antepartum dysglycemia, with those diagnosed with GDM having a 32.8% prevalence of postpartum glucose intolerance compared to only 3.2% in those with normal glucose tolerance.^7^ Additionally, factors such as family history of diabetes and postpartum Body Mass Index (BMI) are predictive of metabolic syndrome and glucose intolerance.^8^^,^^9^ Furthermore, the trajectory of β-cell function and insulin sensitivity post-delivery is distinctly affected by the degree of glucose intolerance experienced during pregnancy, suggesting that early intervention and lifestyle modifications are crucial for managing long-term health outcomes in these women.^9^^,^^10^ Most prior postpartum risk studies emphasize diagnostic OGTT severity or baseline clinical characteristics rather than day-to-day late-pregnancy glycemia captured in routine SMBG.^7^, ^8^, ^9^, ^10^ Whether a pragmatic SMBG-derived time-in-target metric can identify women needing intensified postpartum testing and weight-focused follow-up in routine care therefore remains unclear.^7^, ^8^, ^9^, ^10^

The present study was designed to determine whether the quality of antenatal glycemic control among women with GDM predicts early postpartum dysglycemia and subsequent weight outcomes in routine care. The primary objective was to assess the association between late‑pregnancy glycemic control and postpartum glucose intolerance. Secondary objectives were to evaluate the relationships of antenatal control with PPWR.

## Method

### Study design and setting

The authors conducted a single‑center, prospective, non‑interventional cohort study at Qixia District Hospital to evaluate whether the quality of antenatal glycemic control among women with GDM is associated with postpartum glucose intolerance and PPWR. Consecutive eligible patients were recruited at the time of GDM diagnosis and followed from late pregnancy through 12-months postpartum. Enrollment occurred from May 2021 to April 2024. The study was embedded in routine care without protocol‑mandated treatment changes. The protocol conformed to the Declaration of Helsinki and was approved by the ethics committee of Qixia District Hospital (2025-QX-026). All participants provided written informed consent prior to any study procedures. This prospective observational study was reported in accordance with the Strengthening the Reporting of Observational Studies in Epidemiology (STROBE) statement.

### Participants

Eligible participants were pregnant individuals aged ≥ 18-years with singleton gestations and new GDM diagnoses based on the site’s standard clinical pathways (one‑step 75 *g* IADPSG or guideline‑concordant two‑step approach). Exclusions were pre‑existing type 1 or type 2 diabetes, multiple gestation, major fetal anomaly identified before enrollment, chronic systemic steroid therapy, or medical/social factors precluding postpartum follow‑up. Candidates were identified via diabetes‑in‑pregnancy clinic rosters and obstetric inpatients.

### Antenatal glycemic control

Antenatal glycemia was assessed under routine care using Self‑Monitoring of Blood Glucose (SMBG) and, in a sub‑cohort, Continuous Glucose Monitoring (CGM). Clinical teams trained patients to obtain fasting and postprandial readings ‒ 1 h after meals by default under the institutional GDM pathway, with 2 h postprandial checks used when 1 h sampling was impractical or when the treating clinician judged a later postprandial profile to be more informative for individualized management ‒ at least four times daily on ≥ 5 days/week. For the primary exposure window (28–36 gestational weeks), SMBG data were abstracted from meter memory downloads, clinic‑uploaded logs, or date‑stamped photographs. The prespecified primary exposure was Time‑In‑Target (TIT), the percentage of SMBG readings within pregnancy targets (fasting < 95 mg/dL; 1 h < 140 mg/dL; 2 h < 120 mg/dL when applicable). Secondary descriptors included mean fasting glucose, mean 1 h postprandial glucose, hyperglycemia burden, and glycemic variability. TIT was analyzed as a continuous exposure (per 10% lower) for all primary models. For descriptive and subgroup displays, participants were additionally grouped into empirical quartiles of TIT derived from the observed within‑cohort distribution (equal n per quartile) rather than predefined external cut-points. The resulting boundaries rounded to approximately 60%, 70%, and 80% (Q1 ≥ 80%, Q2 70%–79%, Q3 60%–69%, Q4 < 60%). To enhance clinical interpretability, the authors also prespecified a pragmatic binary threshold of TIT < 60% vs. ≥ 60% for sensitivity analyses and risk communication.

### Outcomes

The primary outcome was postpartum glucose intolerance at 6–12 weeks postpartum, assessed by a laboratory 75 g OGTT using non‑pregnant thresholds (prediabetes: fasting 100–125 mg/dL and/or 2 h 140–199 mg/dL; diabetes: fasting ≥ 126 mg/dL and/or 2 h ≥ 200 mg/dL, with confirmation per clinical practice for single abnormal values). Secondary outcomes were PPWR and substantial PPWR, defined as the difference in kilograms between measured postpartum weight and documented pre‑pregnancy weight, and a binary indicator for PPWR ≥ 5 kg, respectively. Postpartum weights were measured on calibrated digital scales at 6 weeks, 6 months, and 12 months postpartum with light indoor clothing and no shoes. Pre‑pregnancy weight was obtained from the electronic record; if unavailable, the earliest first‑trimester measured weight (≤13 weeks) was used as the proxy baseline. Breastfeeding intensity (exclusive, mixed, none) and brief diet/physical activity indicators were recorded at postpartum visits to contextualize PPWR.

### Covariates

The authors prospectively collected baseline characteristics including ethnicity, parity, maternal age, first‑degree family history of diabetes, smoking, PCOS, pre‑pregnancy BMI (continuous and WHO categories), gestational age at GDM diagnosis, diagnostic OGTT values (fasting, 1 h, 2 h), Institute of Medicine Gestational‑Weight‑Gain (IOM GWG) category (below/within/above), hypertensive disorders of pregnancy, treated thyroid disease, delivery mode, education, and insurance, as well as breastfeeding intensity at 6–12 weeks. For confounding control, the authors defined a Minimally Sufficient Adjustment Set (MSAS) a priori ‒ age, pre‑pregnancy BMI, IOM GWG category, diagnostic OGTT values, parity, treatment modality, and breastfeeding intensity ‒ based on a causal diagram and to maintain ≥10 EPV in the primary model. The authors considered an extended adjustment set that additionally included ethnicity, family history of diabetes, smoking, PCOS, gestational age at diagnosis, education, and insurance in sensitivity analyses. Variables plausibly downstream of the exposure (hypertensive disorders, delivery mode) were not included in the primary model to avoid conditioning on mediators but were explored in sensitivity analyses.

### Data collection, follow‑up, and quality assurance

At GDM diagnosis, baseline demographics, obstetric history, anthropometrics, and diagnostic OGTT values were recorded and SMBG procedures reviewed. Late‑pregnancy visits captured SMBG/CGM exports and treatment changes. Before delivery discharge, the 6–12 week OGTT was scheduled, and automated reminders were issued. Additional reminders were sent for 6‑ and 12‑month weight checks. Clinic scales were verified quarterly with standard weights, and the same device was used longitudinally when possible. SMBG were verified against meter memory when available, with adjudication rules prespecified (meter memory > clinic‑uploaded logs > date‑stamped photographs). De‑identified data were managed in a secure electronic data capture platform with range and logic checks and audit trails; 10% of charts underwent independent source verification. Analysis datasets were version‑controlled and locked prior to modeling.

### Sample size and power

The planned sample (n ≈ 350) provided 80% power (two‑sided α = 0.05) to detect a 15‑percentage‑point absolute difference in postpartum glucose intolerance between better and poorer antenatal control (assumed 20% vs. 35%) while allowing 20% attrition for missed OGTTs. For PPWR, the same sample afforded ≥ 80% power to detect a 1.5 kg mean difference at 12 months, assuming SD = 4.0 kg. The authors enrolled 348 participants, which preserved the planned power given the observed OGTT completion rate.

### Statistical analysis

Descriptive statistics summarized baseline characteristics overall and by TIT quartile using means (SD), medians (IQR), or counts (percentages) as appropriate. Across‑quartile comparisons used ANOVA or Kruskal-Wallis tests for continuous variables and χ^2^ tests for categorical variables, with Benjamini-Hochberg False Discovery Rate (FDR) control applied within families of related tests. The primary dysglycemia analyses were fit among participants with a completed postpartum OGTT; missing covariates were handled with multiple imputation by chained equations (20 datasets), whereas missing OGTT outcomes were not imputed and were instead addressed in sensitivity analyses using complete-case analysis and inverse-probability weighting for OGTT noncompletion. The primary model was used for all prespecified main analyses. Robustness was assessed in an extended model adding ethnicity, family history, smoking, PCOS, gestational age at diagnosis, education, and insurance. The authors also tested a model excluding IOM GWG to evaluate potential overadjustment. Multicollinearity was assessed via variance‑inflation factors (< 2.5 for all models). TIT was modeled continuously as per 10% lower and categorically by quartiles (reference Q1); complementary specifications included mean fasting glucose (per 5 mg/dL higher) and hyperglycemia burden (per 10% higher). Nonlinearity was evaluated using restricted cubic splines with four knots at the 5th, 35th, 65th, and 95th percentiles, displayed as odds ratios relative to TIT = 80%. Effect modification was examined by pre‑pregnancy BMI category (< 25 vs. ≥ 25 kg/m^2^) and treatment modality via interaction terms, with stratum‑specific estimates reported. Model performance was assessed by the Area Under the ROC Curve (AUC) with 95% CIs from 2000 bootstrap resamples, Brier score, calibration slope, and Hosmer-Lemeshow goodness‑of‑fit test. For secondary outcomes, PPWR trajectories at 6-weeks, 6-months, and 12-months were analyzed using linear mixed‑effects models with participant‑level random intercepts and fixed effects for time, continuous TIT, and their interaction, adjusted for the same covariates; substantial PPWR (≥ 5 kg) at 12-months was modeled with multivariable logistic regression. Two‑sided p < 0.05 denoted statistical significance; FDR‑adjusted q‑values accompanied families of related hypotheses. Analyses used R (Version 4.4).

## Result

**Fig. 1:**
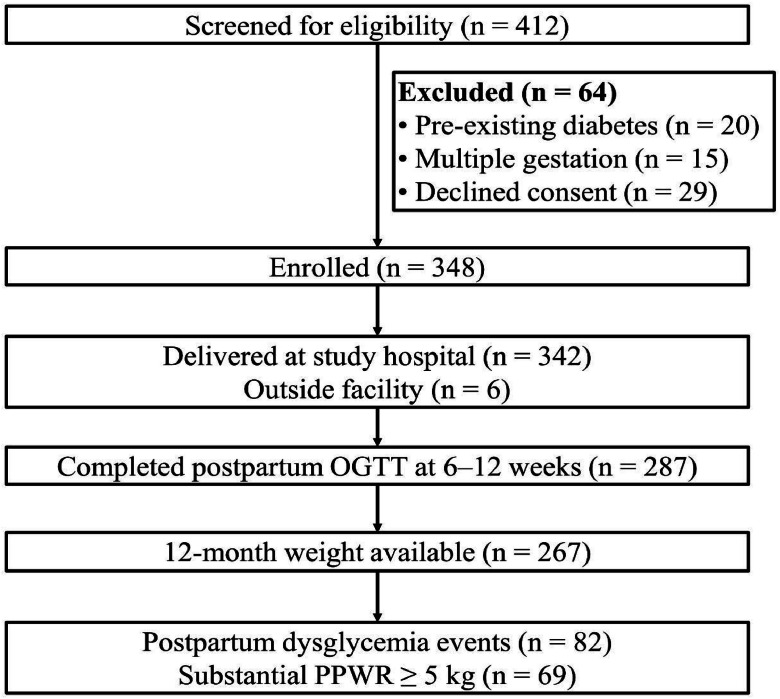
**Participant flow.** Women with Gestational Diabetes Mellitus (GDM) were screened (n = 412), enrolled (n = 348), and followed to delivery at the study hospital (n = 342). The primary analysis set comprised participants completing a 6–12-week Oral Glucose Tolerance Test (OGTT; n = 287, 82.5%). The PPWR analysis set included those with 12-month weight (n = 267, 76.7%). Among OGTT completers, postpartum dysglycemia occurred in 82/287 (28.6%), among those with 12-month weight, PPWR ≥ 5 kg occurred in 69/267 (25.8%).

Among 412 women screened, 348 met eligibility criteria, consented, and were enrolled, and 342 delivered at the study hospital. The primary analysis set comprised 287/348 (82.5%) who completed the 6–12 week OGTT, and 267/348 (76.7%) contributed 12‑month weight for PPWR analyses (Fig. 1). Among those without a completed OGTT (61/348), reasons included missed window (n = 27), no appointment scheduled/logistical barriers (n = 18), relocation outside the network (n = 10), or incomplete sample/laboratory error (n = 6) (Fig. 1). Overall, 82/287 (28.6%) OGTT completers had postpartum glucose intolerance, and 69/267 (25.8%) retained ≥ 5 kg at 12-months.

Baseline characteristics were broadly similar across antenatal control quartiles, with coherent gradients in metabolic severity and treatment intensity (Table 1). Pre‑pregnancy BMI increased from 23.9 ± 3.6 kg/m^2^ in Q1 to 25.8 ± 4.1 kg/m^2^ in Q4 (p = 0.004; FDR q = 0.010), diagnostic OGTT fasting glucose rose from 92.3 ± 8.7 to 98.3 ± 9.9 mg/dL (p = 0.001; FDR q = 0.004), and pharmacologic treatment increased from 23.0% to 51.7% (p < 0.001; FDR q = 0.002). Exceeding Institute of Medicine gestational weight gain recommendations was more common in Q4 than Q1 (33.3%vs. 16.1%, p = 0.002; FDR q = 0.007), whereas parity, hypertensive disorders, and gestational age at GDM diagnosis did not differ meaningfully by quartile (Table 1).

**Table 1:** Baseline characteristics by antenatal glycemic control quartiles (Time-In-Target, TIT).

Characteristic	Overall (n = 348)	Q1 Best (n = 87)	Q2 (n = 87)	Q3 (n = 87)	Q4 Poorest (n = 87)	p-value	FDR q
Maternal age, y	31.1 ± 4.4	30.6 ± 4.2	30.9 ± 4.3	31.3 ± 4.4	31.6 ± 4.7	0.18	0.28
Pre-pregnancy BMI, kg/m^2^	24.8 ± 3.9	23.9 ± 3.6	24.5 ± 3.7	25.1 ± 3.8	25.8 ± 4.1	0.004	0.010
BMI ≥ 25 kg/m^2^	169 (48.6%)	34 (39.1%)	38 (43.7%)	45 (51.7%)	52 (59.8%)	0.006	0.012
Nulliparous	202 (58.0%)	54 (62.1%)	51 (58.6%)	50 (57.5%)	47 (54.0%)	0.64	0.73
Family history of diabetes	119 (34.2%)	24 (27.6%)	28 (32.2%)	31 (35.6%)	36 (41.4%)	0.12	0.21
GA at GDM diagnosis (wk)	26.4 ± 2.1	26.6 ± 2.0	26.5 ± 2.0	26.3 ± 2.1	26.1 ± 2.2	0.20	0.30
Diagnostic OGTT FPG, mg/dL	95.2 ± 9.4	92.3 ± 8.7	94.2 ± 8.9	96.0 ± 9.5	98.3 ± 9.9	0.001	0.004
Diagnostic OGTT 1 h, mg/dL	177 ± 29	169 ± 27	175 ± 28	179 ± 29	184 ± 30	0.003	0.008
Diagnostic OGTT 2 h, mg/dL	156 ± 26	149 ± 24	154 ± 25	157 ± 26	162 ± 27	0.006	0.012
Treatment: pharmacologic	129 (37.1%)	20 (23.0%)	29 (33.3%)	35 (40.2%)	45 (51.7%)	<0.001	0.002
Hypertensive disorders	30 (8.6%)	6 (6.9%)	7 (8.0%)	9 (10.3%)	8 (9.2%)	0.79	0.84
IOM GWG category							
Below	83 (23.9%)	24 (27.6%)	21 (24.1%)	18 (20.7%)	20 (23.0%)	0.44	0.56
Within	178 (51.1%)	49 (56.3%)	48 (55.2%)	43 (49.4%)	38 (43.7%)	0.19	0.28
Above	87 (25.0%)	14 (16.1%)	18 (20.7%)	26 (29.9%)	29 (33.3%)	0.002	0.007
Antenatal SMBG metrics							
TIT (%)	72 ± 14	86 ± 5	74 ± 3	64 ± 3	52 ± 6	<0.001	<0.001
Mean fasting, mg/dL	92 ± 8	85 ± 7	90 ± 7	94 ± 8	98 ± 9	<0.001	<0.001
Mean 1 h postprandial, mg/dL	140 ± 16	125 ± 1 4	135 ± 15	145 ± 16	155 ± 18	<0.001	<0.001
Hyperglycemia burden (%)	27 ± 14	9 ± 6	21 ± 8	32 ± 10	45 ± 13	<0.001	<0.001
SMBG variability (CV, %)	14 ± 4	12 ± 4	13 ± 4	15 ± 4	17 ± 5	<0.001	0.002

**Fig. 2:**
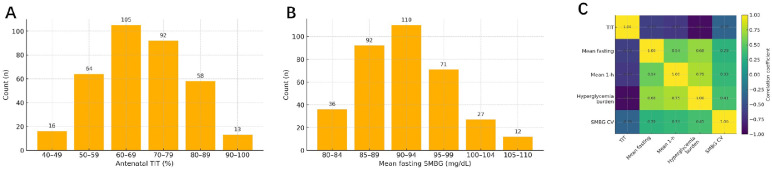
(A) Distribution of antenatal Time‑In‑Target (TIT) during 28–36 weeks. Histogram of the proportion of Self‑Monitoring of Blood Glucose (SMBG) readings within pregnancy targets (fasting < 95 mg/dL; 1 h postprandial < 140 mg/dL; 2 h < 120 mg/dL when used). Prespecified quartiles applied elsewhere: Q1 ≥ 80%, Q2 70%–79%, Q3 60%–69%, Q4 < 60%. (B) Distribution of mean fasting SMBG during 28–36 weeks. Histogram of participant‑level mean fasting glucose (mg/dL) in late pregnancy; values cluster between 85 and 99 mg/dL with a smaller right tail > 100 mg/dL. (C) Spearman correlations among antenatal glycemic metrics. Heatmap of pairwise correlations for TIT, mean fasting glucose, mean 1 h postprandial glucose, hyperglycemia burden (% readings above target), and SMBG Coefficient of Variation (CV). TIT correlates strongly and inversely with hyperglycemia burden (ρ = −0.93) and with mean fasting (ρ = −0.62) and mean 1 h postprandial glucose (ρ = −0.67).

Across the cohort, antenatal TIT centered between 60% and 79%, with 13 participants achieving ≥ 90% and 16 below 50% (Fig. 2A). Mean fasting SMBG clustered between 85 and 99 mg/dL with a smaller right tail > 100 mg/dL (Fig. 2B). Exposure metrics showed strong internal consistency: TIT correlated inversely with hyperglycemia burden (ρ = −0.93) and with mean fasting and mean 1 h postprandial glucose (ρ=−0.62 and −0.67, respectively), while SMBG variability correlated modestly with hyperglycemia burden (ρ = 0.41) and inversely with TIT (ρ = −0.35) (Fig. 2C).

**Fig. 3:**
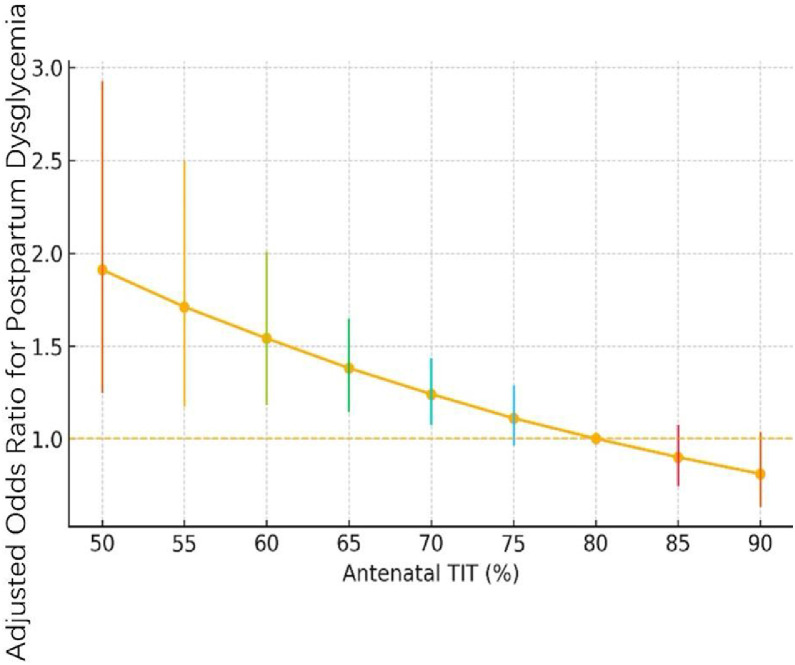
**Adjusted association between antenatal TIT and postpartum dysglycemia.** Odds Ratios (ORs) for any postpartum glucose intolerance at 6–12 weeks are plotted across TIT values relative to TIT = 80% (reference) with 95% confidence intervals. Estimates derive from a multivariable logistic model adjusting for maternal age, pre‑pregnancy BMI, gestational weight‑gain category, diagnostic OGTT values (fasting, 1 h, 2 h), parity, treatment modality (diet vs. pharmacologic), and breastfeeding intensity.

In multivariable models, poorer antenatal control was associated with higher odds of postpartum glucose intolerance (Table 2). Each 10% lower TIT corresponded to an adjusted Odds Ratio (aOR) of 1.24 (95% CI 1.10–1.39; p < 0.001; FDR q = 0.004), and women in Q4 had higher risk than Q1 (aOR = 2.15, 95% CI 1.31–3.53; p = 0.003; FDR q = 0.007). Complementary specifications were concordant, including mean fasting glucose per 5 mg/dL higher (aOR = 1.18, 95% CI 1.05–1.33; p = 0.006; FDR q = 0.011) and hyperglycemia burden per 10% higher (aOR = 1.22, 95% CI 1.08–1.38; p = 0.001; FDR q = 0.004), whereas SMBG variability showed a weaker, non‑significant trend (p = 0.080). Event proportions rose progressively across TIT quartiles among OGTT completers ‒ 18.1% (Q1), 23.6% (Q2), 33.8% (Q3), and 38.9% (Q4) ‒ underscoring a clinically meaningful risk gradient for postpartum follow-up (Table 2). The restricted cubic spline illustrated a graded inverse relationship: relative to TIT 80%, the adjusted odds increased to 1.24 (95% CI 1.08–1.43) at 70% and 1.54 (95% CI 1.19–2.00) at 60%, with wider intervals at extremes (Fig. 3). The primary model demonstrated acceptable performance (AUC = 0.74, 95% CI 0.69–0.78; Brier = 0.19; calibration slope 0.98; Hosmer-Lemeshow p = 0.37) (Table 2).

**Table 2:** Association between antenatal control and postpartum glucose intolerance (primary outcome).

Exposure specification	Adjusted OR (95% CI)	p-value	FDR q
TIT, per 10% lower	1.24 (1.10–1.39)	<0.001	0.004
TIT quartile: Q2 vs. Q1	1.43 (0.82–2.48)	0.200	0.200
TIT quartile: Q3 vs. Q1	1.96 (1.15–3.35)	0.013	0.018
TIT quartile: Q4 vs. Q1	2.15 (1.31–3.53)	0.003	0.007
Mean fasting, per 5 mg/dL	1.18 (1.05–1.33)	0.006	0.011
Hyperglycemia burden, per 10%	1.22 (1.08–1.38)	0.001	0.004
SMBG variability (CV), per 5%	1.17 (0.98–1.40)	0.080	0.093

**Fig. 4:**
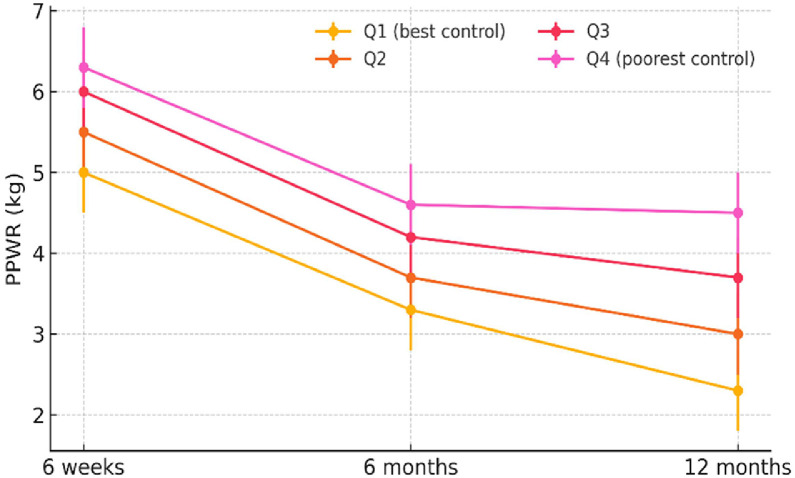
**Postpartum weight‑retention trajectories by antenatal control quartile.** Mean PPWR (kg) ± standard error at 6-weeks (n = 289), 6-months (n = 275), and 12-months (n = 267) stratified by TIT quartiles: Q1 ≥ 80% (best control), Q2 70%–79%, Q3 60%–69%, Q4 < 60% (poorest control). PPWR declines over time in all groups but remains consistently higher with poorer antenatal control.

PPWR declined over time but remained higher with poorer antenatal control (Table 3 and Fig. 4). Overall PPWR was 5.6 ± 4.3 kg at 6-weeks, 4.0 ± 4.2 kg at 6-months, and 3.4 ± 4.1 kg at 12-months; at 12-months, mean PPWR ranged from 2.3 ± 3.7 kg in Q1 to 4.5 ± 4.4 kg in Q4 (Table 3). In adjusted linear models, each 10% lower TIT was associated with higher PPWR of +0.22 kg (95% CI 0.07–0.37; p = 0.004; FDR q = 0.005) at 6 weeks, +0.28 kg (95% CI 0.12–0.44; p = 0.001; FDR q = 0.005) at 6 months, and +0.32 kg (95% CI 0.12–0.52; p = 0.002; FDR q = 0.005) at 12-months (Table 3). For the binary endpoint, each 10% lower TIT increased the odds of substantial PPWR ≥ 5 kg at 12 months (aOR = 1.13, 95% CI 1.02–1.25; p = 0.018; FDR q = 0.021), and Q4 had nearly double the odds versus Q1 (aOR = 1.92, 95% CI 1.11–3.33; p = 0.021; FDR q = 0.021).

**Table 3:** Postpartum Weight Retention (PPWR) outcomes and associations with antenatal control.

A. PPWR (kg) by timepoint and quartile (means ± SD)					
Timepoint	Overall	Q1 Best	Q2	Q3	Q4 Poorest
6-weeks (n = 289)	5.6 ± 4.3	5.0 ± 4.1	5.5 ± 4.2	6.0 ± 4.3	6.3 ± 4.5
6-months (n = 275)	4.0 ± 4.2	3.3 ± 3.9	3.7 ± 4.1	4.2 ± 4.2	4.6 ± 4.3
12-months (n = 267)	3.4 ± 4.1	2.3 ± 3.7	3.0 ± 3.9	3.7 ± 4.1	4.5 ± 4.4

B. Associations (multivariable models, adjusted as in Table 2; FDR within this table)					
Predictor	Outcome & Contrast		Effect (95% CI)	p-value	FDR q
TIT, per 10% lower	PPWR at 6-weeks (kg)		+0.22 (+0.07 to +0.37)	0.004	0.005
TIT, per 10% lower	PPWR at 6-months (kg)		+0.28 (+0.12 to +0.44)	0.001	0.005
TIT, per 10% lower	PPWR at 12-months (kg)		+0.32 (+0.12 to +0.52)	0.002	0.005
TIT, per 10% lower	PPWR ≥ 5 kg at 12-months (aOR)		1.13 (1.02–1.25)	0.018	0.021
TIT quartiles	Q4 vs. Q1: PPWR ≥ 5 kg (aOR)		1.92 (1.11–3.33)	0.021	0.021

Associations for postpartum dysglycemia were stronger among women with pre‑pregnancy BMI ≥ 25 kg/m^2^ (aOR per 10% lower TIT = 1.31, 95% CI 1.12–1.53) than among those with BMI < 25 kg/m^2^ (aOR = 1.16, 95% CI 1.00–1.34), with a statistically significant interaction (p = 0.030; FDR q = 0.040) (Table 4). Effect estimates were similar in diet‑only and pharmacologically treated subgroups without evidence of interaction (p = 0.510), and remained robust when restricting the exposure window to 32–36 weeks (aOR = 1.22, 95% CI 1.08–1.38), excluding pharmacotherapy (aOR = 1.18, 95% CI 1.02–1.37), restricting to complete cases (aOR = 1.25, 95% CI 1.10–1.41), or applying inverse‑probability weighting for OGTT noncompletion (aOR = 1.23, 95% CI 1.09–1.38). In the CGM sub‑cohort, lower time‑in‑range 63–140 mg/dL reproduced the SMBG findings (aOR per 10% lower = 1.28, 95% CI 1.05–1.57), supporting construct validity (Table 4).

**Table 4:** Sensitivity and subgroup analyses for primary outcome (postpartum dysglycemia).

Analysis	n	Adjusted OR (95% CI)	p-value	FDR q
BMI < 25 kg/m^2^	148	1.16 (1.00–1.34)	0.054	0.059
BMI ≥ 25 kg/m^2^	139	1.31 (1.12–1.53)	0.001	0.004
Interaction (BMI strata)	‒	‒	0.030	0.040
Diet-only treatment	181	1.20 (1.04–1.40)	0.016	0.024
Pharmacologic treatment	106	1.27 (1.06–1.53)	0.009	0.018
Interaction (treatment)	‒	‒	0.510	0.510
Exposure window 32–36 weeks	287	1.22 (1.08–1.38)	0.002	0.005
Exclude pharmacologic therapy	181	1.18 (1.02–1.37)	0.034	0.041
Complete-case (no imputation)	276	1.25 (1.10–1.41)	0.001	0.004
Multiple imputation (primary)	287	1.24 (1.10–1.39)	0.0005	0.004
IP-weighted for OGTT noncompletion	348	1.23 (1.09–1.38)	0.0015	0.005
CGM sub-cohort (n = 72): TIR 63–140, per 10% lower	72	1.28 (1.05–1.57)	0.015	0.024

## Discussion

In this single‑center cohort, postpartum dysglycemia was common, and poorer antenatal glycemic control independently identified women at higher risk; those in the poorest‑control quartile had roughly double the risk versus the best‑control quartile.

These findings show that poorer antenatal glycemic control, operationalized as lower SMBG time‑in‑target across 28–36 weeks, was independently associated with higher odds of postpartum glucose intolerance at 6–12 weeks and with greater 12‑month PPWR, with a graded pattern from the best to the poorest control quartile and stronger effects among women with pre‑pregnancy BMI ≥ 25 kg/m^2^. This dose–response aligns with longitudinal work demonstrating that the severity of gestational dysglycemia predicts early postpartum dysglycemia and metabolic syndrome after GDM.^11^ β‑cell function also declines within the first postpartum year in women with recent glucose intolerance in pregnancy, plausibly linking gestational hyperglycemia to early deterioration in glucose tolerance.^12^ Pregnancy CGM literature supports pregnancy‑specific targets and shows that higher late‑gestation TIR is associated with better perinatal outcomes.^13^, ^14^, ^15^ The present CGM sub‑analysis recapitulated the SMBG signal, suggesting construct validity of “time‑within‑goal” as a clinically meaningful exposure. The PPWR gradient the authors observed is consistent with cohorts showing that PPWR tracks adverse lipids, blood pressure, and glucose trajectories for up to five years after delivery, reinforcing PPWR as an early cardiometabolic risk marker rather than a cosmetic outcome.^16^^,^^17^ Mechanistically, gestational hyperglycemia reflects heightened insulin resistance and β‑cell vulnerability. After delivery, persistent positive energy balance, especially in those with overweight/obesity, sustains PPWR and worsens insulin resistance. Conversely, lactation improves maternal glucose utilization and is associated with lower T2DM incidence and lower PPWR after GDM,^18^^,^^19^ supporting the adjustment for breastfeeding intensity and the observed BMI.

Postpartum risk is not determined by antenatal glycemia alone. Contraceptive choice, particularly progestin-only methods, may influence postpartum weight or diabetes risk.^20^^,^^21^ Depressive symptoms screened with the Edinburgh Postnatal Depression Scale (EPDS) or an equivalent instrument and short or poor-quality sleep may hinder postpartum self-management and are associated with less favorable postpartum weight outcomes.^22^^,^^23^ Postpartum blood pressure and intercurrent conditions such as treated thyroid disease should likewise be reviewed when interpreting early postpartum metabolic risk; women with prior GDM have increased early postpartum hypertension risk.^24^

Clinically, antenatal control can function as a risk stratifier to prioritize postpartum OGTT completion and early lifestyle support: ADA Standards of Care recommend a 75 g OGTT at 4–12 weeks postpartum and periodic screening thereafter, with OGTT preferred over A1C in the early postpartum period.^25^ Implementation strategies should match risk: programs that perform the OGTT during the delivery hospitalization or offer very‑early testing can improve uptake, although results from very‑early testing should be interpreted alongside the standard 4–12 week OGTT.^26^^,^^27^ These approaches, coupled with digital outreach and appointment scheduling before discharge, can counter the well‑documented shortfalls in postpartum testing.^26^, ^27^, ^28^, ^29^ The present data suggest that women with TIT < 60% (poorest quartile in thiscohort) constitute a high‑risk group for targeted outreach and counseling, although external validation is needed before adopting a universal threshold. Because poorer antenatal control also predicted higher PPWR, postpartum lifestyle interventions adapted from the Diabetes Prevention Program are timely, with trials showing prevention of weight gain or reduced PPWR within 12-months.^30^^,^^31^ For women with impaired glucose tolerance after GDM, metformin is an evidence‑based preventive option comparable to lifestyle in this subgroup.^32^^,^^33^ Finally, risk‑stratified pathways must be coupled with equity‑minded implementation, given persistent disparities in postpartum screening by race/ethnicity and insurance.^28^^,^^29^ Using antenatal control as a trigger within integrated systems can help direct resources where they are most needed.^27^^,^^28^

This study’s limitations include single‑center generalizability, potential misclassification from variable SMBG adherence despite meter‑memory verification, residual confounding inherent to observational designs, and attrition for postpartum assessments, which the authors addressed using multiple imputation, complete‑case checks, and inverse‑probability weighting, though some selection bias may remain. The CGM analysis was limited to a sub‑cohort, and lactation/diet/physical activity were measured briefly rather than with comprehensive instruments. The authors also did not systematically capture postpartum contraceptive method, EPDS-based mental health screening, sleep measures, or postpartum blood pressure, and treated thyroid disease was available only from routine clinical documentation rather than standardized postpartum reassessment. Future work should validate these risk gradients and candidate thresholds across multi‑center and more diverse populations, test intervention strategies that use antenatal control patterns to trigger tailored care, and integrate continuous CGM‑derived time‑in‑range as a standardized exposure to refine risk stratification and decision support in routine obstetric‑diabetes pathways.

In routine care, the quality of antenatal glycemic control in gestational diabetes identified women at higher risk of both early postpartum dysglycemia and greater 12‑month postpartum weight retention. Although observational and not causal, these results support using antenatal control metrics as a practical risk signal to prioritize postpartum 75 g OGTT completion, structured follow-up, and tailored early weight‑management support that includes lactation promotion and structured lifestyle programs. Embedding equity‑minded implementation strategies and evaluating cost‑effectiveness will be essential to translate antenatal control signals into measurable improvements in postpartum metabolic health.

## Ethics approval and consent to participate

The protocol conformed to the Declaration of Helsinki and was approved by the ethics committee of Qixia District Hospital (Approval number: 2025-QX-026). All participants provided written informed consent prior to any study procedures.

## Data availability

Data sets generated during the current study are available from the corresponding author on reasonable request.

## Clinical trial number

Not applicable.

## Consent to publish declaration

Not applicable.

## Authors’ contributions

The authors confirm contribution to the paper as follows: Study conception and design: Y.J.; Data collection: Y.J.; Analysis and interpretation of results: Y.J.; Draft manuscript preparation: Y.J. All authors reviewed the results and approved the final version of the manuscript.

## Funding

No funding.

## Declaration of competing interest

The authors declare no conflicts of interest.
